# Research and exploration of quality control indicators for nutritional therapy in critically ill patients—a multicenter before-and-after controlled study

**DOI:** 10.3389/fnut.2024.1359409

**Published:** 2024-07-18

**Authors:** Yanhua Li, Youquan Wang, Bin Chen, Zhe Zhang, Dong Zhang

**Affiliations:** ^1^Department of Critical Care Medicine, The First Hospital of Jilin University, Changchun, China; ^2^Department of Nephrology, The First Hospital of Jilin University, Changchun, China

**Keywords:** nutritional support, enteral nutrition, achievement rate, quality control, quality evaluation criteria

## Abstract

**Objective:**

To evaluate and explore the feasibility of using quality control indicators for nutritional therapy in critically ill patients as quality evaluation criteria.

**Methods:**

This study focused on intensive care unit (ICU) critically ill patients and conducted a cross-sectional investigation of nutritional therapy quality control indicators (the proportion of patients with application of enteral nutrition pump, nutritional risk assessment rate, the proportion of patients start enteral nutrition within 48 hours, and caloric and protein target achievement rate on 7th day) in 13 hospitals in Jilin Province. After training according to the critical patients nutrition related guidelines and the latest literatures, a second cross-sectional investigation was conducted. Then, analyze the improvement of quality control indicators of the nutritional therapy before and after the training, thus evaluating the feasibility of using these quality control indicators as nutritional therapy quality evaluation criteria in critical patients.

**Results:**

(1) A total of 631 patients were included before and after training, with a data acquisition rate of 97.3% for enteral nutrition pumps usage and complete data collection for the remaining nutritional risk assessment rate, start enteral nutrition proportion of patients within 48 h, and caloric and protein target achievement rate on 7th day. (2) The nutritional risk assessment rate before and after training was 88.2% vs. 94.8%, with a *P*-value of 0.003. The proportion of patients start enteral nutrition within 48 h before and after training was 65.1% vs. 75.4%, with a *P*-value of 0.039; and protein target achievement rate on 7th day before and after training was 64.6% vs. 79.6%, with a *p*-value of 0.015. These five indicators as quality evaluation criteria are relevant to the current developments in nutritional therapy and consistent with the national conditions of China. The proportion of patients with application of enteral nutrition pump before and after training was 70.1% vs. 79.4%, with a *p*-value of 0.065, and the caloric target achievement rate on 7th day before and after training was 73.4% vs. 83.9%, with a *p*-value of 0.062, and there was no statistical difference between the two groups.

**Conclusion:**

The five quality control indicators for nutritional therapy in critically ill patients are clinically feasible and can be used as quality evaluation criteria for nutritional therapy in critically ill patients.

## 1 Introduction

Nutrition support is an important aspect of critical patient management, and current research suggests that the smooth implementation of early enteral nutrition can improve the prognosis of critically ill patients (1). However, there is still a significant gap between the theory and clinical practice of critical care nutrition, especially in primary hospitals where the medical level is uneven. How to improve the tolerance of enteral nutrition and standardize the feeding protocol of medical staff has become an important issue facing nutrition support. Therefore, the quality control of nutritional therapy in critically ill patients is very important, However, there are no relevant studies on the quality control of nutritional support. In order to promote the continuous improvement of the quality of critical care medicine, Professor Chen Dechang led the establishment of the “Critical Illness Digestive Working Group” in May 2022, and formulated the “Critical Care Nutrition Quality Control Indicators.” This study investigated the feasibility of implementing critical illness nutrition quality control indicators by training 13 primary hospitals in Jilin Province on nutrition guidelines and related literature by investigating changes in five indicators of “critical illness nutrition quality control indicators” before and after training. The five indicators of critical illness nutrition quality control indicators are proportion of patients receiving enteral nutrition pump infusion, nutritional risk assessment rate, the proportion of patients start enteral nutrition within 48 hand caloric/protein target achievement rate on 7th day.

## 2 Methods

### 2.1 Overall design

This is a multicenter before-and-after controlled study. This study aimed to validate the clinical feasibility by analyzing the changes of five indicators between before and after the training on nutrition guidelines and related literature (2) at 13 primary hospitals in Jilin Province. The specific feeding protocol can be seen in Supplementary Figure S1. The five indicators include the proportion of patients receiving enteral nutrition pump infusion, nutritional risk assessment rate, the proportion of patients initiating enteral nutrition within 48 h, and caloric/protein target achievement rate on the 7th day. The first retrospective cross-sectional study was conducted from 0:00 at November 7, 2022 to 24:00 at November 14, 2022. On November 19, 2022, we conducted training on standardized feeding procedure based on the latest domestic feeding protocol (2). Subsequently, the same five quality control indicators were investigated again from February 1, 2023 to February 28, 2023 in the same 13 primary hospitals.

Before the start of this research project, we through several meetings to collect voluntary to join this research unit and physician, a total of recruited 48 doctors involved in 13 hospitals in Jilin province.

### 2.2 Inclusion and exclusion criteria

All patients admitted to the ICU during the investigation period were included in the study, except for patients whose physicians did not agree to participate in the training or provide data collection.

### 2.3 Data collection

We collected the patient’s data including age, gender, height, weight, whether there is a primary gastrointestinal injury, Acute Physiology and Chronic Health Evaluation II,(APACHEII); Sequential Organ Failure Assessment (SOFA), whether a nutritional risk assessment was performed, whether an enteral nutrition pump was used, whether enteral nutrition was started within 48 h, the reason for not initiating enteral nutrition within 48 h, whether the calorie and protein targets were achieved on the 7th day, and the incidence of feeding intolerance (FI) during the first 7 days in ICU. ICU Length of Stay, ICU Mortality and so on.

This study has been approved and informed consent by the Ethics Committee of the First Hospital of Jilin University (AF-IRB-030-06.). All methods in this research were performed in accordance with the relevant guidelines and regulations.

### 2.4 Statistical methods

This cross-sectional study was an observational study. Statistical analysis was performed using SPSS 24.0 software. Normally distributed measurement data were represented as mean ± standard deviation (SD), non-normally distributed measurement data was represented as median (interquartile range), and the counting data were represented as rate. The homogeneity test of variance was performed for normally distributed measurement data, and the *t*-test was performed for comparison of mean values between two groups of normally distributed data with homogeneity of variance. The *t*-test was performed for comparison of mean values between two groups conforming to normal distribution but with unequal variances, and the rank sum test was performed for measurement data conforming to non-normal distribution. The comparison of each observation index before and after promotion was performed using paired chi-square tests. For a total sample size of 40 or more, where all theoretical frequencies were greater than or equal to 5, Pearson’s chi-square test was applied. If one theoretical frequency was less than or equal to 5 and greater than or equal to 1 among a total sample size of 40 or more, continuity correction would be performed. If two theoretical frequencies were less than or equal to 5 and greater than or equal to 1 among a total sample size of 40 or more, Fisher’s exact test would be used. *p* < 0.05 indicates statistical significance.

## 3 Results

### 3.1 General data statistics of enrolled patients

The first stage enrolled a total of 246 patients, and the second stage enrolled 385 patients. Their basic information is described in Table 1.

**Table 1:** General characteristics of included patients.

	Before training (*n* = 246)	After training (*n* = 385)	*P*
Age (years, mean ± *SD*)	63.55 ± 16.05	62.94 ± 15.74	0.64
Male, *n* (%)	59.8%	61.0%	0.75
PAGI, *n* (%)	46.3%	50.6%	0.33
APACHEII score (mean ± *SD*)	18.97 ± 8.53	19.13 ± 8.12	0.81
SOFA (median, IQR)	6[3,9]	5[3.8]	0.35
ICU length of stay (days, median, IQR)	4[3,8]	5[3,9]	0.78
ICU mortality, *n* (%)	13.8%	10.6%	0.26

PAGI, primary acute gastrointestinal injury; APACHE II, Acute Physiology and Chronic Health Evaluation II; SOFA, Sequential Organ Failure Assessment; SD, standard deviation; IQR, interquartile range; ICU, intensive care unit.

### 3.2 Five quality control indicators before and after training

#### 3.2.1 Proportion of patients receiving enteral nutrition pumps infusion

The proportion of patients receiving enteral Nutrition pumps infusion in the ICU is calculated as the number of patients receiving enteral nutrition pumps infusion divided by the total number of patients receiving enteral nutrition therapy during the same period. This rate reflects the standardization of enteral nutrition pumps usage in the ICU. The data for this analysis were mainly obtained from medical record system, which includes not only medical orders but also nursing records.

During the first stage, a total of 110 cases received enteral nutrition, among which information on the use of an enteral nutrition pump was available for 107 cases, accounting for 97.3%. In addition, there were three instances where it remained ambiguous whether an enteral nutrition pump was utilized, based on the information extracted from medical record system. Among the 107 patients utilizing enteral nutrition pumps, 75 patients were found to be utilizing enteral nutrition pumps while 32 patients were not, resulting in the proportion of patients receiving enteral nutrition pumps infusion is 70.1%.

During the second stage, a total of 209 cases received enteral nutrition, among which 166 cases used an enteral nutrition pumps and while 43 cases did not, resulting in the proportion of patients receiving enteral nutrition pumps infusion is 79.4%.

Chi-square test was performed to compare the proportion of patients receiving enteral nutrition pump infusion between the two stages before and after the training, with a *p*-value of 0.065 showing no statistical difference. Please refer to Table 2 for details.

**Table 2:** Comparison of quality control indicators before and after training, as well as incidence of feeding intolerance.

Variables	Total *n*	Before training	After training	*P*-value
Using ENP, *n* (%)	316	75(70.1%)	166(79.4%)	0.065
NRA has been conducted, *n* (%)	631	217(88.2%)	365(94.8%)	0.003
Start EN within 48 h, *n* (%)	337	95(65.1%)	144(75.4%)	0.039
The caloric goal has been met on the 7th day, *n* (%)	216	58(73.4%)	115(83.9%)	0.062
The protein goal has been met on the 7th day, *n* (%)	216	51(64.6%)	109(79.6%)	0.015
The incidence of FI, *n* (%)	462	48(27.9%)	94(32.4%)	0.35

ENP, enteral nutrition pump; NRA, nutritional risk assessment; EN, enteral nutrition; FI, feeding intolerance.

#### 3.2.2 The rate of nutritional risk assessment

The rate of nutritional risk assessment refers to the proportion of patients admitted to the ICU who undergoes nutritional risk assessment application of nutritional risk assessment tool NRS2002 or NUTRIC score according to the guidelines recommended (3) within 24 h of admission, out of the total patients treated in the ICU during the same period. It reflects the nutritional risk status of ICU patients.

The results of the nutritional risk assessment are stored in electronic or paper-based nursing records or electronic medical records, and can be obtained by reviewing the medical records or nursing documentation to determine whether the patients admitted to the ICU have undergone nutritional risk assessment.

In the first stage, there were a total of 246 cases. Among these cases, 217 cases underwent nutritional risk assessment and 29 cases did not. The rate of nutritional risk assessment was 88.2%. Among those assessed, 81 cases were identified as low nutritional risk, 110 cases as high nutritional risk, and 26 cases were categorized as other.

In the second stage, there were a total of 385 cases. Among these cases, 365 cases underwent nutritional risk assessment and 20 cases did not. The rate of nutritional risk assessment was 94.8%. Among those assessed, 98 cases were identified as low nutritional risk, 225 cases as high nutritional risk, and 42 cases were categorized as other.

The results also showed that nearly all enrolled patients undergo nutritional risk assessment using NRS ratings, only two cases used the NUTRIC score. The nutritional risk assessment rate was compared between the two stages before and after the training using a chi-square test, *p*-value is 0.003, indicating a statistically significant difference. After the training, the rate of nutritional risk assessment was higher. Please refer to Table 2 for details.

#### 3.2.3 The proportion of patients start enteral nutrition within 48 h

The proportion of patients start enteral nutrition within 48 h refers to the number of patients who initiate enteral nutrition within 48 h of admission to the ICU, divided by the total number of patients who stayed in the ICU for more than 48 h during the same period and there were no contraindications to EN. This information can be obtained by reviewing medical record system.

In the first stage, there were a total of 246 cases. One hundred eighty-two cases stayed in the ICU for more than 48 h. Among these cases, there were 36 cases with contraindications for enteral nutrition (12 cases with unstable hemodynamics, 8 cases with inaccessible distal feeding access in patients with intestinal fistula, 5 cases with severe acidosis or hypoxia, 5 cases with uncontrolled gastrointestinal bleeding, 2 cases with gastric retention exceeding 500 mL every 6 h, 2 cases with confirmed intestinal obstruction, and 2 cases with abdominal compartment syndrome). There were 146 cases without contraindications for enteral nutrition. Of these, 95 cases started enteral nutrition within 48 h, while 51 cases did not receive enteral nutrition within 48 h (26 cases were post-gastrointestinal surgery, 13 cases were multiple trauma patients, and 12 cases were severe acute pancreatitis). The proportion of patients start enteral nutrition within 48 h was 65.1%.

**Figure 1:**
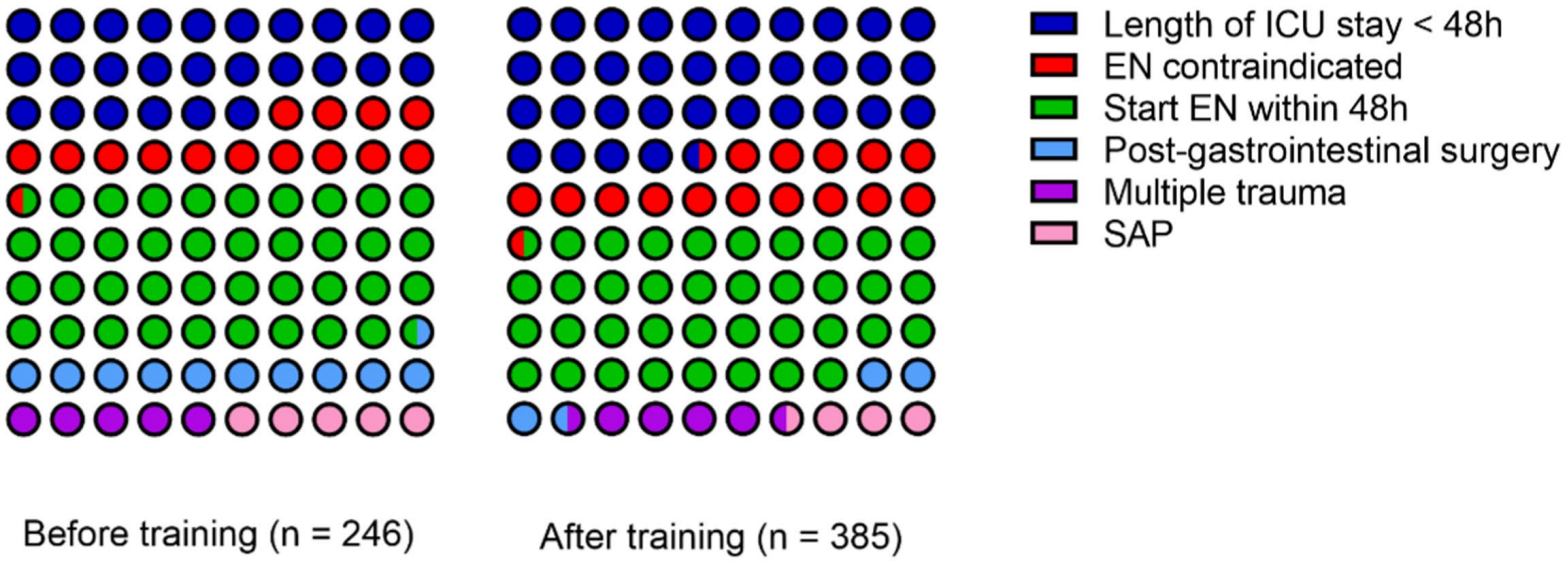
Specific reasons for the failure to initiate enteral nutrition within 48 h.

In the second stage, there were a total of 385 cases. Among these, 253 cases stayed in the ICU for more than 48 h. Among these cases, there were 62 cases with contraindications for enteral nutrition (22 cases with unstable hemodynamics, 6 cases with inaccessible distal feeding access in patients with intestinal fistula, 14 cases with severe acidosis or hypoxia, 6 cases with uncontrolled gastrointestinal bleeding, 5 cases with gastric retention exceeding 500 mL every 6 h, 5 cases with confirmed intestinal obstruction, and 4 cases with abdominal compartment syndrome). There were 191 cases without contraindications for enteral nutrition. Among of these, 144 cases started enteral nutrition within 48 h, while 47 cases did not receive enteral nutrition within 48 h (15 cases were post-gastrointestinal surgery, 18 cases were multiple trauma patients, and 14 cases were severe acute pancreatitis). The proportion of patients start enteral nutrition within 48 h was 75.4%. The specific reasons for the failure to initiate enteral nutrition within 48 h in both stages can be referred to in Figure 1.

A chi-square test was performed to compare the proportion of patients start enteral nutrition within 48 h before and after the training. The *p*-value was 0.039, indicating statistically significant differences. After the training, the proportion of patients start enteral nutrition within 48 h was higher. Please refer to Table 2 for details.

**Figure 2:**
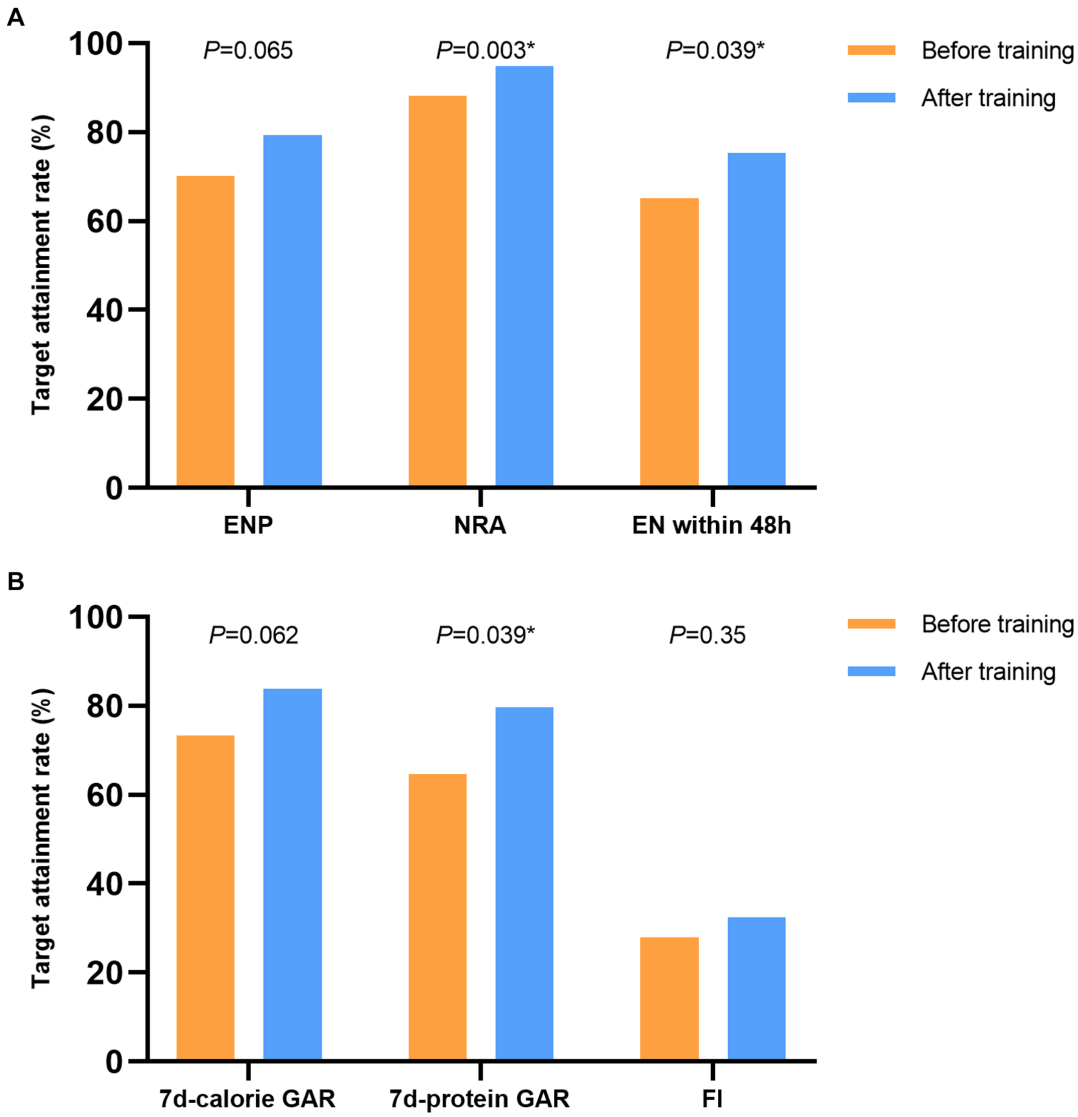
The results of the nutrition therapy before and after the training. **(A)** The process indicators for the nutrition therapy. **(B)** The results of the nutrition therapy. ENP, enteral nutrition pump; NRA, nutritional risk assessment; EN, enteral nutrition; GAR, goal achievement rate, FI, feeding intolerance.

The proportion of patients receiving enteral nutrition pumps infusion, the rate of nutritional risk assessment and the proportion of patients start enteral nutrition within 48 h are the process indicators for the quality control. The results of before and after the training are showed in Figure 2A.

#### 3.2.4 The caloric target achievement rate on 7th day

The caloric target achievement rate on the 7th day refers to the proportion of patients meeting the calorie target on the 7th day. The calculation formula involves the ratio of patients who have attained over 70% of their prescribed calorie intake, determined by a simplified weight-based equation (25 kcal/kg/day), to the total number of ICU-admitted patients with a hospitalization duration exceeding 7 days within the same timeframe. This reflects the calorie target achievement status of ICU patients. The information can be obtained by reviewing the electronic medical record system, including medical orders and nursing records.

In the first stage, there were a total of 246 cases. One hundred seven cases stayed in the ICU for 7 days or more. Among these, there were 28 cases with oral feeding. We exclude patients with ICU stays less than 7 days and those who are able to take oral intake. Seventy-nine patients were included in the calculation. Among these, 58 patients achieved the calorie target, while 21 patients did not. The caloric target achievement rate on 7th day was 73.4%.

In the second stage, there were a total of 385 cases. Among them, 195 cases stayed in the ICU for 7 days or more and 58 cases with oral feeding. We exclude patients with ICU stays less than 7 days and those who are able to take oral intake. One hundred thirty-seven patients were included in the calculation. Among these, 115 patients achieved the calorie target, while 22 patients did not. The caloric target achievement rate on 7th day was 83.9%.

A chi-square test was conducted to compare the caloric target achievement rate on 7th day between the two stages before and after the training. The *p*-value was 0.062, indicating no statistically significant difference. The data results are detailed in Table 2.

#### 3.2.5 The protein target achievement rate on 7th day

The protein target achievement rate on 7th day refers to the proportion of patients meeting the protein target on the 7th day. The calculation formula involves the ratio of patients who have attained 1.3 g/kg.d within 7 days of ICU admission based on guidelines published in 2019 (4), to the total number of ICU-admitted patients with a hospitalization duration exceeding 7 days within the same timeframe. This reflects the protein target achievement status of ICU patients, and the information can be obtained by reviewing electronic medical record system.

In the first stage, there were a total of 246 cases. Among them, 107 cases stayed in the ICU for 7 days or more. There were 28 cases with oral feeding. We also exclude patients with ICU stays less than 7 days and those who are able to take oral intake, a total of 79 patients were included in the calculation. Among these, 51 patients achieved the protein target, while 28 patients did not. The protein target achievement rate on 7th day was 64.6%.

In the second stage, there were 385 cases. Among them, 195 cases stayed in the ICU for 7 days or more. There were 58 cases with oral feeding, we exclude patients with ICU stays less than 7 days and those who are able to take oral intake. A total of 137 patients were included in the calculation. Among these, 109 patients achieved the protein target, while 28 patients did not. The protein target achievement rate on 7th day was 79.6%.

According to the provided information, the chi-squared test was conducted between the two stages before and after the training, and the *p*-value was 0.015, indicating a statistical difference between the two stages. The protein target achievement rate on the seventh day was higher after the training. This suggests that the implementation of the standardized protocol may have a positive impact on improving the protein target achievement rate. The data results are detailed in Table 2.

### 3.3 The incidence of feeding intolerance (FI)

The incidence of feeding intolerance (FI) has a certain correlation with the prognosis of critically ill patients. FI diagnostic criteria, as proposed by the 2012 ESICM guidelines (5), was applied in this study. FI was defined as the inability to tolerate EN due to any clinical reasons, such as vomiting, high gastric residual volume (a single residual volume ≥200 mL), diarrhea (≥3 loose or liquid stools per day, stool weight exceeding 200–250 g/day or 250 mL/day), gastrointestinal bleeding, or enterocutaneous fistula. In addition, FI was considered present if enteral nutrition could not achieve a minimum of 20 kcal/kg/day via the enteral route within 72 h of initiation, or if enteral feeding had to be discontinued for any clinical reasons (6).

In the first stage, 172 cases can collected the data of whether feeding intolerance occurs. Among them, 48 patients experienced feeding intolerance and 124 patients did not experience feeding intolerance. The FI incidence rate is 27.9%.

In the second stage, 290 cases can collected the data of whether feeding intolerance occurs. Among them, 94 patients experienced feeding intolerance and 196 patients did not experience feeding intolerance. The FI incidence rate is 32.4%.

According to the provided information, the chi-squared test was conducted between the two stages before and after the training, and the *p*-value was 0.35, indicating no statistically significant difference. The data results are detailed in Table 2.

The calorie target achievement rate on 7th day and the protein target achievement rate on 7th day are the results indicators for the quality control. The incidence of feeding intolerance is the indicator of feeding tolerance and adverse reactions. The results of before and after the training are showed in Figure 2B.

## 4 Discussion

In the field of medical science, medical quality control, also known as healthcare quality control (QC), has gained increasing prominence and importance with the country’s advancement and the continuous improvement of healthcare standards. As early as 2006, scholars proposed a series of measures to enhance quality and safety control in intensive care units (ICUs) (7, 8). In 2015, the former National Health and Family Planning Commission officially released the “Medical Quality Control Indicators for Critical Care Medicine (2015 edition).” In 2020, the Critical Care Medicine Professional Quality Control Center of the National Health Commission once again issued a statement to promote the establishment of quality control indicators for 15 key diseases or techniques, including “ICU gastrointestinal nutrition technology” (9). Therefore, under the leadership of Professor Chen Dechang, the “Critical Care Digestive Work Group” engaged in in-depth discussions for a period of 2 years and formulated a set of quality control indicators specifically for critical care nutrition. These indicators are designed based on the principles of being target-driven, measurable, and aligned with the specific national context of China. They consist of five indicators, including process indicators and outcome indicators. Nutritional risk assessment rate, the proportion of patients with application of enteral nutrition pump and the start enteral nutrition proportion of patients within 48 h can be used as process indicators. The calorie target achievement rate and the protein target achievement rate on 7th day can be used as outcome indicators for the quality evaluation of nutrition therapy in critically ill patients. All these indicators are recommended with explicit guidance in recent guidelines.

As the sole representative of the Critical Care Digestive Work Group in Jilin Province, our organization aimed to evaluate and deliberate on the practicality of clinical implementation of the five selected critical care nutrition quality control indicators. Our objective was to present clinical data for national critical care nutrition quality control and explore relevant indicators for evaluating the quality of nutrition therapy in critically ill patients. In the context of the “National Critical Care Disease Nutrition Treatment Improvement Quality Month” initiative, we conducted a survey on nutrition evaluation-related indicators within the province. Our objective was to further investigate metrics for assessing the quality of nutrition therapy in critically ill patients. Enteral nutrition is the preferred method of nutrition for ICU patients (3). There are many advantages to enteral nutrition in critically ill patients, including reducing inflammation, restoring muscle function, providing micronutrients and macronutrients, maintaining intestinal integrity, and promoting insulin sensitivity (10). Multiple studies have also shown that early enteral nutrition can reduce the incidence of infectious complications and mortality (11, 12). Therefore, many guidelines recommend early initiation of enteral feeding (within 24–48 h) for most ICU patients (3, 4, 13). However, critically ill patients often have gastrointestinal dysfunction, leading to enteral feeding intolerance and consequences of malnutrition (14). Malnutrition has negative effects on quality of life and patient outcomes, and can lead to increased healthcare costs (15, 16). Its incidence can be as high as 12.6–52% (17), and research has shown that the incidence of hospital-acquired malnutrition can reach 25.9%, which is associated with longer hospital stays, higher readmission rates at 6 months, and may be related to higher rates of complications and infections (18). The ratio of EN/EN + PN is negatively correlated with mortality (19). Therefore, many guidelines recommend screening for nutritional risk and continuous feeding for ICU patients, as continuous pump feeding can increase the tolerance of enteral nutrition (4, 20–22).

We conducted a survey of 13 primary hospitals in Jilin Province to collect data on the proportion of patients receiving enteral nutrition pumps infusion, nutritional risk assessment rates, start enteral nutrition proportion of patients within 48 h, and the calorie target achievement rate on 7th day and the protein target achievement rate on 7th day for a total of 631 patients. In the vast majority of cases, relevant information could be found in patient medical records and doctor’s orders, resulting in good data completeness. This indicates that these five indicators are measurable.

According to our survey results, the rate of nutritional risk assessment was 88.2%, the proportion of patients with application of enteral nutrition pump was only 70.1%, the proportion of patients start enteral nutrition within 48 h for ICU patients was 65.1%, the calorie target achievement rate on 7th day was 73.4%, and the protein target achievement rate on 7th day was 64.6%. These findings suggest that there is a significant gap between clinical practices and guidelines in ICU nutritional support therapy in our province, which is consistent with the conclusions of another large-scale multicenter survey on enteral nutrition in China in recent years (23). Therefore, it implies the need for a further improvement in the standardization of clinical practices to raise the level of nutritional support therapy for patients. In this study, we also conducted training on critical care nutrition-related guidelines and literature (2–4, 13) for 13 ICUs in Jilin Province. Subsequently, we re-evaluated the pertinent critical care nutrition indicators and observed enhancements in all 5 quality control measures.

The survey results showed that nearly all enrolled patients undergo nutritional risk assessment using NRS ratings, only two cases used the NUTRIC score. This indicates that NRS is more practical than NUTRIC as a quality control indicator for nutritional risk assessment. Moreover, statistical analysis compared the data before and after training. There are significant statistical differences in the rates of nutritional risk assessment, the proportion of patients start enteral nutrition within 48 h, and the protein target achievement rate on 7th day, but no statistical difference in the proportion of patients receiving enteral nutrition pumps infusion and the calorie target achievement rate on 7th day.

We analyzed the reasons for these findings. We further investigated the pump-to-bed ratio at each center. The mean pump-to-bed ratio was 0.61, which did not change in the two phases of the study. It suggested that the limited number of enteral nutrition pumps in the department may have hindered the improvement of the proportion of patients receiving enteral nutrition pumps infusion. So, this may suggest that we should add the “pump to bed” ratio as a structural indicator of nutrition quality control. In regard to the calorie target achievement rate on 7th, recent studies have suggested that the optimal target for the calorie intake is still unclear (21, 24, 25). In fact, some studies have even suggested that overfeeding may result in adverse nutritional effects through mechanisms such as autophagy and ketosis (26, 27). Improvements in critical care nutrition quality evaluation indicators before and after training have demonstrated that the standardized nutrition support process training and promotion effectively improved the standardization of nutrition support therapy in our province. Furthermore, two rounds of quality control indicator surveys have demonstrated that these 5 quality control evaluation indicators for critical care nutrition align with the national conditions in China. The incidence of feeding intolerance (FI) can result in disruptions or delays in EN implementation, ultimately leading to compromised energy acquisition, malnutrition, and prolonged hospitalization, with potential subsequent increased mortality. The gastrointestinal tract of severely ill patients is fragile and there may be a risk of feeding intolerance (FI) (28–30). Contrary to our expectation, the incidence of feeding intolerance did not decrease after the promotion of standardized nutritional support. The likely reasons leading to these outcomes may be attributed to the increased the proportion of patients start enteral nutrition within 48 h and protein target achievement rate on 7th day by the 7th day following training.

This study is the first multicenter study on quality control indicators for nutritional support therapy. However, this study also has limitations. For example, some enrolled patients were fed orally. Since we could not accurately calculate their protein and caloric intake, we excluded them when calculating caloric and protein attainment on the 7th day. That might have some impact on the actual attainment rate of them. Furthermore, there are many patients who are fasting due to contraindications for enteral nutrition. We have excluded them when calculating the proportion of patients with application of enteral nutrition pump and the proportion of patients start enteral nutrition within 48 h, which has reduced our sample size. Moreover, the scope of this study is limited to 13 primary hospitals in Jilin Province. The investigation time is short and the sample size is limited, so it may not be applicable nationwide or even internationally. Further multicenter studies with expanded scope are needed to further validate the findings.

## 5 Conclusion

The five indicators of nutritional risk assessment rate, the proportion of patients receiving enteral nutrition pumps infusion, the proportion of patients starting enteral nutrition within 48 h, caloric and protein target achievement rate on the 7th day can serve as robust quality control indicators for patients undergoing intensive nutritional therapy in critically ill patients. Moreover, we propose incorporating the “pump to bed” ratio as a structural indicator to assess the quality of nutrition therapy in critically ill patients.

## Data availability statement

The original contributions presented in the study are included in the article/Supplementary material, further inquiries can be directed to the corresponding author.

## Ethics statement

The studies involving humans were approved by Medical Ethics Committee of the First Hospital of Jilin University. The studies were conducted in accordance with the local legislation and institutional requirements. The Ethics Committee/Institutional Review Board waived the requirement of written informed consent for participation from the participants or the participants’ legal guardians/next of kin because there was no intervention for the patients in this study, the data collected were the medical records of the patients during their treatment, without personal information.

## Author contributions

YL: Investigation, Writing – original draft. YW: Data curation, Methodology, Visualization, Writing – original draft. BC: Writing – original draft, Writing – review & editing. ZZ: Data curation, Investigation, Writing – original draft. DZ: Conceptualization, Funding acquisition, Resources, Supervision, Writing – review & editing.
