# Supplementary figures and images for: Research and exploration of quality control indicators for nutritional therapy in critically ill patients—a multicenter before-and-after controlled study
Source: Front Nutr. 2024 Jul 18;11:1359409. doi: 10.3389/fnut.2024.1359409 (PMC11291468; doi:10.3389/fnut.2024.1359409)

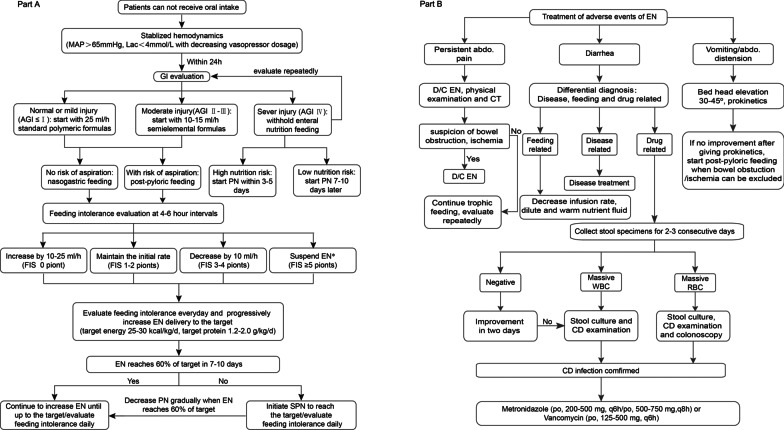

Supplement: Supplementary file 1 [file Image_1.JPEG]
